# Tracheostomy and long-term mortality in ICU patients undergoing prolonged mechanical ventilation

**DOI:** 10.1371/journal.pone.0220399

**Published:** 2019-10-02

**Authors:** Raphaël Cinotti, Sebastian Voicu, Samir Jaber, Benjamin Chousterman, Catherine Paugam-Burtz, Haikel Oueslati, Charles Damoisel, Anaïs Caillard, Antoine Roquilly, Fanny Feuillet, Alexandre Mebazaa, Etienne Gayat, On behalf of FROG-ICU investigators

**Affiliations:** 1 Department of Anaesthesia and Critical Care, Hôpital Laennec, University Hospital of Nantes, Saint-Herblain, France; 2 Department of Medical and Toxicological Intensive Care, Hôpital Lariboisière, Assistance Publique Hôpitaux de Paris, Paris, France; 3 Department of Anesthesia and Critical Care Department B, Saint Eloi Teaching Hospital, University Hospital of Montpellier, France; 4 INSERM UMR 942 “Biocanvass”, Hôpital Lariboisière, Assistance Publique Hôpitaux de Paris, France; 5 Department of Anaesthesia and Critical Care, Hôpital Lariboisière, Assistance Publique Hôpitaux de Paris, Paris, France; 6 Department of Anesthesia and Critical care department, Hôpital Beaujon, Assistance Publique des Hôpitaux de Paris, Clichy, France; 7 Department of Anesthesia and Critical care department, Hôpital Saint-Louis, Assistance Publique des Hôpitaux de Paris, Paris, France; 8 Laboratoire UPRES EA 3826 « Thérapeutiques cliniques et expérimentales des infections », University hospital of Nantes, Bio-Ouest, Institut de la Recherche, Nantes, France; 9 INSERM UMR 1246 –SPHERE « Methods in Patient-Centered Outcomes and Health Research », Institut de la Recherche, Nantes, France; University of Notre Dame Australia, AUSTRALIA

## Abstract

**Introduction:**

In critically ill patients undergoing prolonged mechanical ventilation (MV), the difference in long-term outcomes between patients with or without tracheostomy remains unexplored.

**Methods:**

Ancillary study of a prospective international multicentre observational cohort in 21 centres in France and Belgium, including 2087 patients, with a one-year follow-up after admission. We included patients with a MV duration ≥10 days, with or without tracheostomy. We explored the one-year mortality with a classical Cox regression model (adjustment on age, SAPS II, baseline diagnosis and withdrawal of life-sustaining therapies) and a Cox regression model using tracheostomy as a time-dependant variable.

**Results:**

29.5% patients underwent prolonged MV, out of which 25.6% received tracheostomy and 74.4% did not. At one-year, 45.2% patients had died in the tracheostomy group and 51.5% patients had died in the group without tracheostomy (p = 0.001). In the Cox-adjusted regression model, tracheostomy was not associated with improved one-year outcome (HR CI95 0.7 [0.5–1.001], p = 0.051), as well as in the model using tracheostomy as a time-dependent variable (OR CI 95 1 [0.7–1.4], p = 0.9).

**Conclusions:**

In our study, there was no statistically significant difference in the one-year mortality of patients undergoing prolonged MV when receiving tracheostomy or not.

**Trial registration:**

NCT01367093

## Introduction

Prolonged weaning from mechanical ventilation (MV) in the Intensive Care Unit (ICU) is associated with high mortality [1,2], and few strategies have been recently identified to improve outcome in these patients. Tracheostomy has been proposed more than 20 years ago to improve weaning and seems to be more frequently utilised in recent years [3]. However, randomized-controlled trials, as well as observational studies [4], did not demonstrate better long-term outcomes in patients receiving early (<day 7) as compared to late tracheostomy (>day 14) [5,6]. The realization of tracheostomy is thus not currently recommended for the weaning of mechanical ventilation [7].

The comparison between a tracheostomy versus a no-tracheostomy policy in patients undergoing prolonged MV or weaning has been poorly explored [8]. Moreover, long-term outcomes are less described and are probably of major interest, knowing the long-term morbidity and mortality in these severe ICU patients [9]. We hypothesized that tracheostomy can improve the outcomes of patients with prolonged MV. We elaborated an ancillary analysis of the FROG-ICU study [10] in patients receiving prolonged MV duration with or without tracheostomy and studied the one-year mortality in these two groups. We also studied the evolution of the quality of life during the follow-up.

## Material and methods

This is an ancillary study of the FROG-ICU study (NCT01367093).[10] This study was approved by ethics committee (Comité de la Protection des Personnes—Ile de France IV, IRB n°00003835 and Comission d’Ethique Biomédicale Hospitalo-Facultaire de l’hôpital de Louvain, IRB n°B403201213353). Patients or next-of-kin provided written consent for participation in the study. The FROG-ICU is a multi-centre international observational study performed in 21 ICUs (medical, surgical and mixed) in 14 university hospital in France and Belgium, which included 2087 patients with a one-year follow-up after ICU admission. Briefly, patients were included in case of invasive mechanical ventilation (MV) ≥ 24 hours and/or treatment with vasoactive treatment (except for Dopamine). Non-inclusion criteria were a Glasgow coma score ≤ 8, brain death or persistent vegetative state, pregnancy, transplantation in the previous 12 months, moribund state and the lack of social security coverage.

### Study population

In this ancillary study, we analysed patients with prolonged MV (≥10 days) [6,11]. Patients with early tracheostomy were included as long as the overall duration of mechanical duration was ≥10 days. Exclusion criteria were unknown duration of MV, tracheostomy upon ICU admission, unknown timing of tracheostomy and patients without MV during ICU stay. The threshold of 10 days was upheld because in an international multi-centric observational study in patients with Acute Respiratory Distress Syndrome (ARDS) [11], patients with the most severe pattern of ARDS underwent at least 9 days of MV and displayed higher mortality [6].

### Primary outcome

The primary outcome was the one-year all-cause mortality in patients undergoing prolonged MV (≥10 days) with or without tracheostomy.

### Secondary outcome

The secondary outcome was the evolution of quality of life (Short-Form 36 (SF-36) survey), symptoms of post-traumatic stress disorder (Impact of Event Scale–Revisited (IES-R) [12]) and symptoms of anxiety and depression (Hospital Anxiety Depression Scale (HADS) [12]), between patients with or without tracheostomy in the first year after ICU admission.

### Data-collection

Clinical and biological data were recorded at admission and during the ICU stay: age, gender, age-adjusted Charlson score, SAPS II, history of cardio-vascular and respiratory diseases, cause of ICU admission, in-ICU complications (transfusion, renal replacement therapy, withdrawal of life-sustaining therapies (WLST)), timing of tracheostomy, duration of MV, ICU length of stay, oxygen at ICU discharge, facility after hospital discharge, outcomes. A follow-up was performed during the first year after ICU admission, at 3, 6 and 12 months. Quality of life evaluation was performed at 3,6 and 12 months with the SF-36, IES-R and HADS.

### Statistical analysis

Continuous data are expressed as mean (±standard deviation) or median [quantile] and compared with the Student t-test or Mann-Whitney test whenever appropriate. Nominal data are expressed as N(%) and compared with the Chi2 or Fisher exact test whenever appropriate. The primary outcome was the evolution of the one-year mortality in patients undergoing prolonged MV (≥10 days) with or without tracheostomy. Kaplan Meier curves for cumulative mortality were elaborated during the one-year period. Comparison of survival were performed with a Log-Rank test. Several models were elaborated. First, we performed a classical Cox regression model adjusted on age, Simplified Acute Physiological Score II (SAPS II), diagnosis on admission and withdrawal of life-sustaining therapies (WLST). Diagnosis on admission were categorized: neurologic cause, respiratory failure, sepsis/septic shock and others. The time zero of the Kaplan Meier curve was set at day-10 after admission (prolonged MV duration). Second, owing to the survival bias patients with mortality in the first weeks, might not receive tracheostomy. We thus performed another Cox regression model, adjusted on age, SAPS II, cause of ICU admission, WLST and transformed tracheostomy as a time-dependent variable. Third, given the observational nature of the data, the treatment allocation (tracheostomy) was not randomly assigned in the studied population. We used propensity-score matching to reduce the risk of bias due to confounders and study more accurately the link between tracheostomy and outcome [13]. Each patient treated with tracheostomy was matched to one untreated control (prolonged MV) with a similar propensity score. Variables included in the propensity score model were selected from the available baseline variables based on known associations between factors and exposure (prolonged MV duration) (age, gender, neurologic cause of admission, acute respiratory failure at admission, SAPS II, co-morbidities (age-adjusted Charlson score), red blood pack transfusion, in-ICU renal replacement therapy and in-ICU vasoactive treatment. Treated and not treated patients were matched according to the nearest neighbour approach within a calliper width of 0.1. To assess the balance of covariates between the two groups before and after propensity-score matching, mean standardized differences (MSD) were used. A mean standardized difference *<*10% was considered to support the assumption of balance between groups [14]. In this matched sample, we performed a Cox regression model adjusted on tracheostomy, chronic obstructive pulmonary disorders and centre.

Because of the major impact of WLST on outcome, we performed a sensitivity analysis in the sub-group of patients without WLST. In this subgroup of patients, Kaplan Meier curves for cumulative mortality were elaborated during the one-year period. Outcome was compared with a Log-Rank test, and a Cox regression model adjusted on age, SAPS II and diagnosis on admission.

A 2-way ANOVA analysis was performed when analysing the evolution of SF-36, IES-R and HADS over time and the differences between the two groups, with an interaction test. In case the patient died during the follow-up, he/she was excluded from the quality of life analysis. We did not impute a zero value to the quality of life data in this setting [15]. The Hazard Ratio (HR) are presented with their 95% Confidence of Interval (CI).

Patients without a known duration of MV were not included in this study, and no multiple imputation analysis were performed. All statistical tests were two-sided. A *p* value <0.05 was considered statistically significant. Statistical analyses were performed with R Studio version 1.0.136, with the *Matching* and *Matchit* packages (The “R” Foundation for Statistical Computing, Vienna, Austria).

## Results

**Fig 1:**
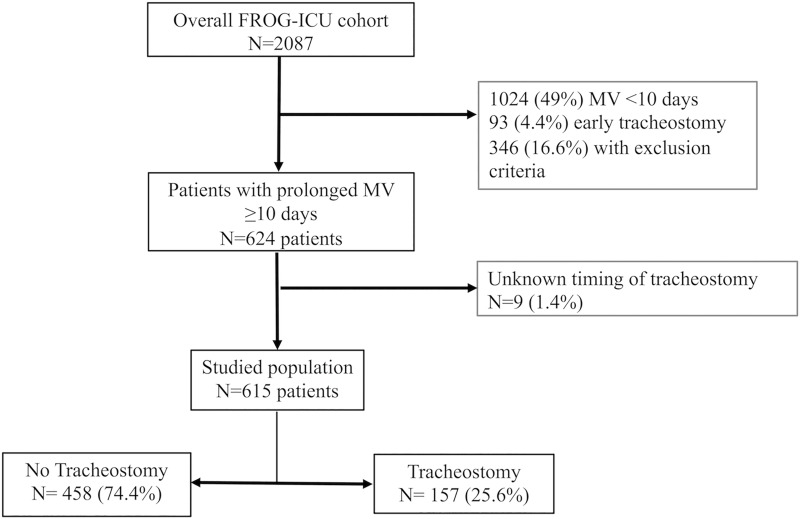
Flowchart of the study. Flowchart of patients with prolonged mechanical ventilation (≥ 10 days), with or without tracheostomy, included in the FROG-ICU sub-study.

Out of the 2087 included in the FROG-study, 1024 (49%) patients had a duration of MV shorter than 10 days, 93 (4.4%) patients had early tracheostomy and 346 (16.6%) presented exclusion criteria. We thus analysed 157 (25.6%) patients with tracheostomy (≥10 days) and 458 (74.4%) without (Fig 1). Tracheostomy was performed with a median timing of 20 [16–27] days.

### Study population

Full demographic data are displayed in Table 1. Patients with tracheostomy were younger than patients with prolonged MV (59 (±15) years vs. 63 (±16) years, p = 0.005), had lower SAPS II score (52 (±20) vs 47 (±21), p = 0.005) and less co-morbidities assessed with the Charlson score (3 [1–4] vs. 3 [1–5], p = 0.04). There were no significant differences in baseline causes of admission with patients without tracheostomy (p = 0.06). During their ICU stay, patients receiving tracheostomy had less WLST (13 (8.2%) vs. 84 (18.3%), p = 0.001). The median timing of WLST was 18 [12–27] days. In patients with tracheostomy, the median timing of WLST was 44 [29–48] days. In the no-tracheostomy group, the median timing of WLST was 16 [11–23] days.

**Table 1:** Baseline characteristics of patients undergoing prolonged mechanical ventilation with or without tracheostomy.

	No Tracheostomy N = 458	Tracheostomy N = 157	*p*
Timing of tracheostomy	_	20 [16–27]	_
Age	63 (±16)	57 (±15)	0.005
Gender M/F	297 (64.8) / 161 (35.2)	110 (70.1) / 47 (29.9)	0.2
SAPS II	52 (±20)	47 (±21)	0.005
SOFA score	8 [5–11]	8 [5–10]	0.9
Charlson	3 [1–5]	3 [1–4]	0.04
GCS on admission	14 [4–15]	15 [9–15]	0.04*
Reason for ICU admission			0.06
Resuscitated cardia arrest	35 (8%)	10 (6%)	
Respiratory failure	97 (21%)	21 (13%)	
Neurological	62 (14%)	32 (20%)	
Sepsis/Septic shock	123 (27%)	43 (27%)	
Other	141 (31%)	51 (33%)	
Cardio-vascular co-morbidities			
Hypertension	229 (50%)	60 (38%)	0.009
Diabetes mellitus	92 (20%)	23 (15%)	0.1
Ischemic myocardiopathy	16 (4%)	2 (1%)	0.2^£^
Chronic vascular disease	40 (9%)	17 (11%)	0.4
Respiratory co-morbidities			
COPD	72 (16%)	19 (12%)	0.3
Active smoking	133 (29%)	40 (26%)	0.4
Other significant co-morbidities			
Chronic kidney failure	50 (11%)	18 (12%)	0.9
Stroke	25 (6%)	7 (5%)	0.8
Cognitive dysfunction	10 (2%)	1 (0.6%)	0.3^£^
Loss of autonomy	17 (4%)	4 (3%)	0.6^£^
ICU discharge			
RBC transfusion during ICU	273 (60%)	106 (68%)	0.08
ICU LOS > 20 days	216 (47%)	151 (96%)	<0.05
Duration of MV	15 [12–20]	20 [12–26]	0.001*
SBP < 100 mmHg at discharge	116 (25%)	33 (21%)	0.1
110 ≤ SBP ≤ 140 mmHg at discharge	140 (31%)	65 (41%)	
SBP > 140 mmHg at discharge	58 (13%)	24 (15%)	
Temperature < 37°C at discharge	121 (26%)	49 (31%)	0.8
Protein < 60g.L^-1^ at discharge	101 (22%)	28 (18%)	0.1
Life Sustaining Therapy Withdrawal	84 (18%)	13 (8%)	0.001
Tracheostomy at ICU discharge	_	119 (76%)	**_**
Hospital Discharge			
Oxygen therapy	16 (5%)	8 (5%)	0.5
Tracheostomy at hospital discharge	_	96 (61%)	_
Facility transfer			0.01
Ward	40 (9%)	17 (11%)	
Home	111 (24%)	39 (25%)	
Step down unit	2 (0.4%)	7 (4%)	
Rehabilitation centre	51 (11%)	31 (20%)	
Palliative care	1 (0.2%)	_	
Other	74 (16%)	29 (18%)	
O_2_ therapy during follow-up	3 (0.6%)	1 (0.6%)	_

GCS: Glasgow Coma Score. COPD: Chronic Obstructive Pulmonary Disease. MV: Mechanical Ventilation. Numeric data are analysed with student or wilcoxon* test accordingly. Categorical data are analysed with Chi2 test or Fisher test^£^ accordingly.

### Primary outcome

**Fig 2:**
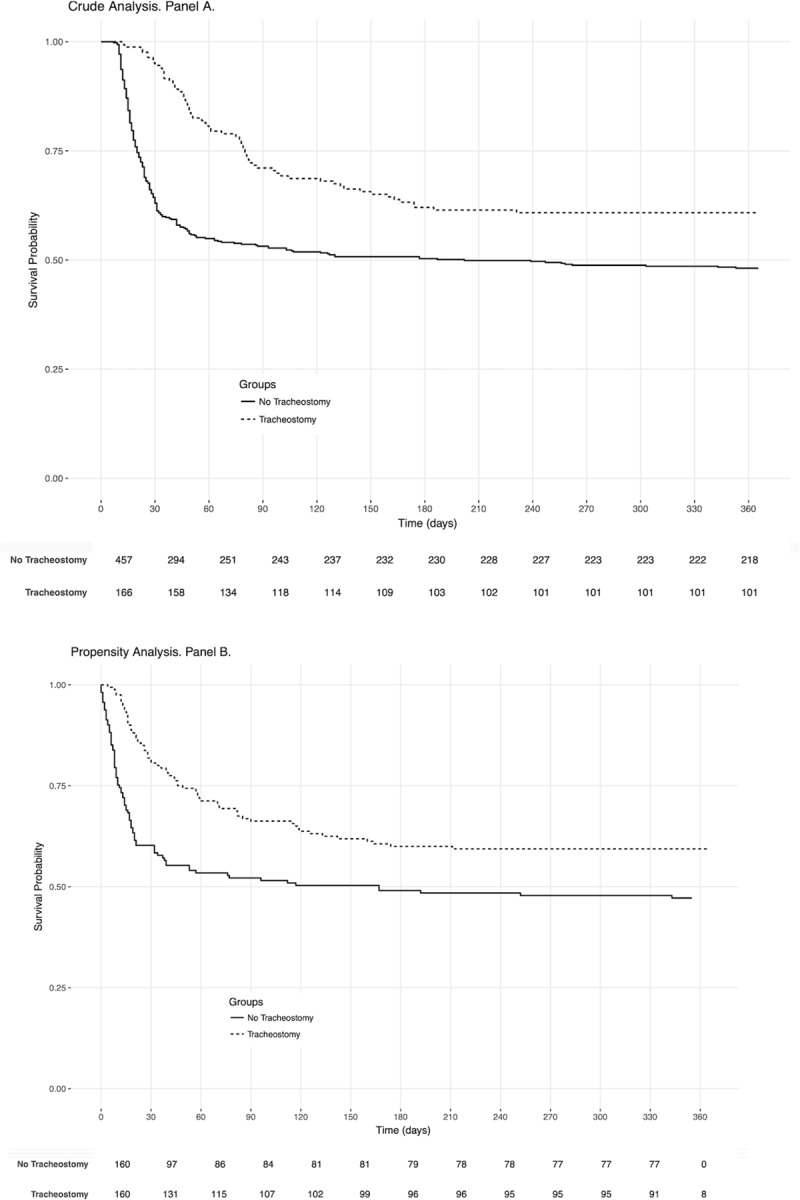
One-year survival curves in patients with prolonged mechanical ventilation duration (≥10 days) with or without tracheostomy, in crude analysis and in propensity-analysis. Kaplan-Meier curves of one-year mortality in crude analysis (p = 0.001, log-rank test, HR CI95 0.7 [0.5–1.001], p = 0.051, Panel A) and in a propensity-score analysis (HR CI95 0.6 [0.5–0.9], p = 0.02, Panel B).

One-year after ICU admission, 71 (45.2%) patients had died in the tracheostomy group and 236 (51.5%) in the no-tracheostomy group (p = 0.001, log-rank test) (Fig 2, Panel A). In the Cox-adjusted regression model, adjusted on age, baseline cause of admission, SAPS II score and WLST, there was no significant difference between tracheostomy and no- tracheostomy on the one-year mortality (HR CI95 0.7 [0.5–1.001], p = 0.051) (Table 2).

**Table 2:** Cox-adjusted analysis regression model on one-year mortality in patients with prolonged MV with or without tracheostomy.

Factors	HR CI95%	p
Tracheostomy	0.7 [0.5–1.001]	0.051
Age 60–80	_	_
Age <60	0.7 [0.5–1.1]	0.1
Age ≥80	1.7 [1.09–2.9]	0.02
SAPS II	1.01 [1.005–1.02]	0.0009
Acute respiratory failure	1.1 [0.6–1.9]	0.6
Neurologic cause	0.8 [0.4–1.5]	0.4
Sepsis/septic shock	1.2 [0.7–1.9]	0.4
WLST	5.6 [4.1–7.8]	<0.0005

SAPS II: Simplified Acute Physiological score. WLST: Withdrawal of Life-Sustaining Therapies.

We matched 150 patients in each group with a propensity score (S1 Table). In this matched sample, there was an association between improved outcome in the group with tracheostomy compared to patients with no- tracheostomy (HR CI95 0.6 [0.5–0.9], p = 0.02) (Fig 2, Panel B).

The Cox regression model, using tracheostomy as a time-dependent variable, showed no significant association between the one-year outcome and the use of tracheostomy (OR CI 95 1 [0.7–1.4], p = 0.9). In a sensitivity analysis, we excluded patients who underwent WLST in the ICU. There was no significant difference between patients with or without tracheostomy on the one-year mortality regarding the Log-Rank test (p = 0.08) and the Cox regression model (HR CI95 0.74 [0.5–1.09], p = 0.1)(S1 Fig).

### Secondary outcome

**Fig 3:**
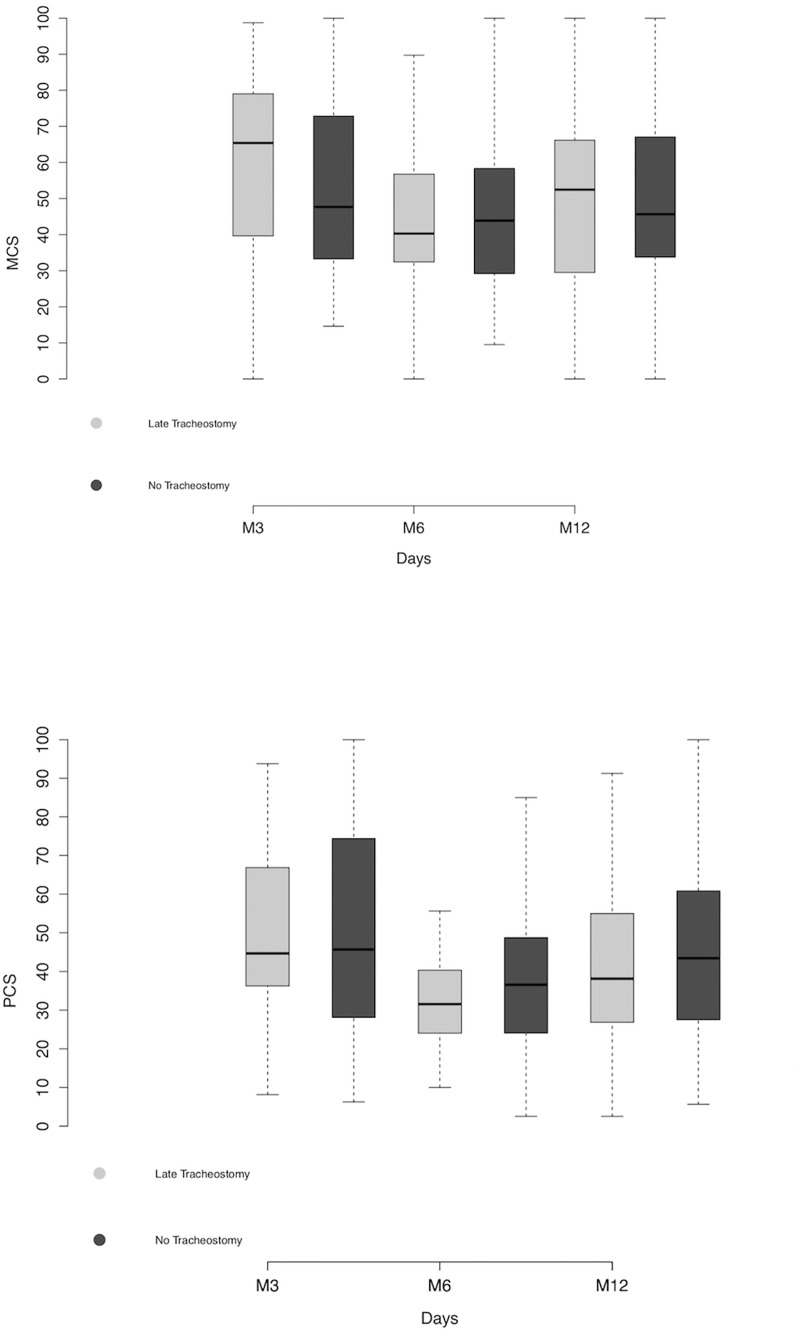
Evolution of the MCS and PCS component of the SF-36 at 3, 6 and 12 months after ICU admission in patients with tracheostomy or no tracheostomy and prolonged mechanical ventilation. The quality of life was measured by the Short Form-36 (SF-36) at 3, 6 and 12 months after ICU admission. SF-36 is a made of a mental (Mental Component Scale, MCS) and of a physical (Physical Component Scale, PCS) component. Each component ranges from 0 (poor quality of life) to 100 (upper quality of life). There is no significant difference in the MCS (Left panel, p = 0.5) and in the PCS (Right panel, p = 0.4) between the two groups. Two-way ANOVA.

We compared the evolution of the quality of life during the one-year follow-up in survivors with tracheostomy or without tracheostomy. At 3 months, we gathered quality of life questionnaires in 44 (12.8%) patients who underwent tracheostomy and in 53 (28%) patients without tracheostomy. At one-year, we gathered data in 38 (24.2%) patients who underwent tracheostomy and in 48 (11.5%) patients without. Regarding quality of life assessment, there were no differences between the mental and physical components of the SF-26 between the two groups, during the first year of follow-up (Fig 3, S2 Table). Regarding anxiety/depression symptoms and post-traumatic stress disorders, there were no differences between the groups regarding the HADS and IES-R scales respectfully (S2 Table).

## Discussion

Our study shows that in ICU patients undergoing prolonged MV, tracheostomy is not associated with an improved one-year outcome. In patients with prolonged MV and weaning [1], few recent therapeutic progresses have been made. In a recent international observational study of Acute Respiratory Distress Syndrome (ARDS) patients [16], a duration of MV > 9 days was observed in patients with the most severe ARDS pattern and these patients displayed a higher mortality rate than patients with mild-to-moderate ARDS. As previously described [3,6], we have thus selected 10 days as the cut-off for the definition of prolonged MV.

In a recent meta-analysis pooling 11 studies [17]studying the association between early and late tracheostomy on patients’ outcomes, the authors found a decrease in the ICU length of stay and in the duration of sedation, in the early tracheostomy group. However, there was no difference in the in-hospital mortality. In another meta-analysis [18], there was no statistically significant difference regarding the one-year mortality in patients receiving early tracheostomy, but only 3 studies assessed this outcome.

Several differences must be underlined with the current work. First, early tracheostomies with low duration of MV were not included in our analysis and we focused in patients undergoing protracted MV with or without tracheostomy. This strategy has been poorly explored in the current literature and moreover, we herein display long-term outcomes, which has been rarely document in this topic. In spite of negative results, we bring new light on the effects of tracheostomy in patients with prolonged MV.

There was no difference in the quality of life assessed by the SF-36 between the 2 groups during the one-year follow-up. However, these results should be cautiously interpreted, since there is a selection bias since only 34.6% of the patients included in this sub-study provided quality of life questionnaires during the follow-up. During post-critical care illness follow-up, a high number of patients are unfortunately lost in the process. In a monocentric study [19] evaluating the self-reported quality of life, 60% of patients could performed a complete follow-up. In another 2-year follow-up performed in a critical care pediatric population [20], around 55% of children underwent complete neuropsychological assessment. This underlines the complexity of performing exhaustive follow-up after critical care illness. In spite of an excellent follow-up regarding the primary outcome in our study, there quality of life assessment in the first year after ICU admission remains disappointing, and implies a selection bias. Therefore, it is very difficult to assess the impact of tracheostomy on the quality of life in survivors with prolonged MV duration.

There is a statistically significant imbalance in WLST between both groups, which is of major importance regarding outcome. When excluding these patients, our sensitivity analysis showed the same trend of the relative effect of tracheostomy on outcome. This sensitivity analysis displays a loss of power, which could explain, at least in part, why these results are not statistically significant. Moreover, WLST were performed after the realisation of tracheostomy and are probably not a confounding factor of the effects of tracheostomy.

Our study has limitations. First, this is an observational study and therefore our result cannot reflect causation between tracheostomy and outcomes. Also, the reason of tracheostomy was not recorded, which implies a selection bias. There was a significant imbalance between the 2 groups regarding WLST. This point hampers solid conclusions regarding the long-term effects of tracheostomy in severe critically ill patients. Second, we have missing data regarding the duration of MV and we did not perform multiple imputation analysis. The quality of life questionnaires were retrieved by postmail during follow-up. This explains the missing values and also implies selection bias. Our proportion of patients receiving prolonged MV could seem high compared to others [1], but the differences in inclusion criteria as well as the definition of prolonged weaning could explain these discrepancies. Finally, the divergent results between the Cox regression model and the propensity-score analysis could be explained by the difference in the number of variables considered for adjustment and propensity matching. However, taking into account the survival bias, the Cox regression model using tracheostomy as a time-dependent variable, did not display significance.

## Conclusion

In this international multi-centre observational study, tracheostomy was not associated with improved one-year mortality in ICU patients receiving prolonged MV (≥10 days). Methodological issues such as selection bias, renders the results difficult to generalize. Randomized-Controlled Trials are needed before drawing definitive conclusions, but interventional studies in ICU patients with prolonged MV remain challenging [8].

## Supporting information

S1 Fig One-year survival curves in patients undergoing prolonged mechanical ventilation duration (≥10 days) with or without tracheostomy, in the sub-group of patients without WLST. In the subgroup of patients without withdrawal of life sustaining therapies, there was no significant difference between patients with or without tracheostomy. Log-Rank test, p = 0.08. Cox regression model adjusted on age, SAPS II and diagnosis upon admission: HR CI95 0.74 [0.5–1.09], p = 0.1.
(TIFF)

S1 Table Patients matched in the Tracheostomy group and in the prolonged MV group without tracheostomy. SAPS: Simplified Acute Physiological Score. RBC: red blood cell.
(DOCX)

S2 Table Long-term quality of life in patients with or without tracheostomy. During the first year of follow-up, there was no statistically significant difference regarding the Mental and Physical components of the SF-36 between both groups (p = 0.9 and 0.9 respectively). There is no statistically significant difference in IES-R, Anxiety symptoms (HADS scale) or Depression symptoms (HADS scale) between the 2 groups (respectively p = 0.9, 0.2, 0.5), or evolution over time (respectively p = 0.1, p = 0.4, p = 0.3). Two-way ANOVA.
(DOCX)
