# Supplementary figures and images for: Tracheostomy and long-term mortality in ICU patients undergoing prolonged mechanical ventilation
Source: PLoS One. 2019 Oct 2;14(10):e0220399. doi: 10.1371/journal.pone.0220399 (PMC6774500; doi:10.1371/journal.pone.0220399)

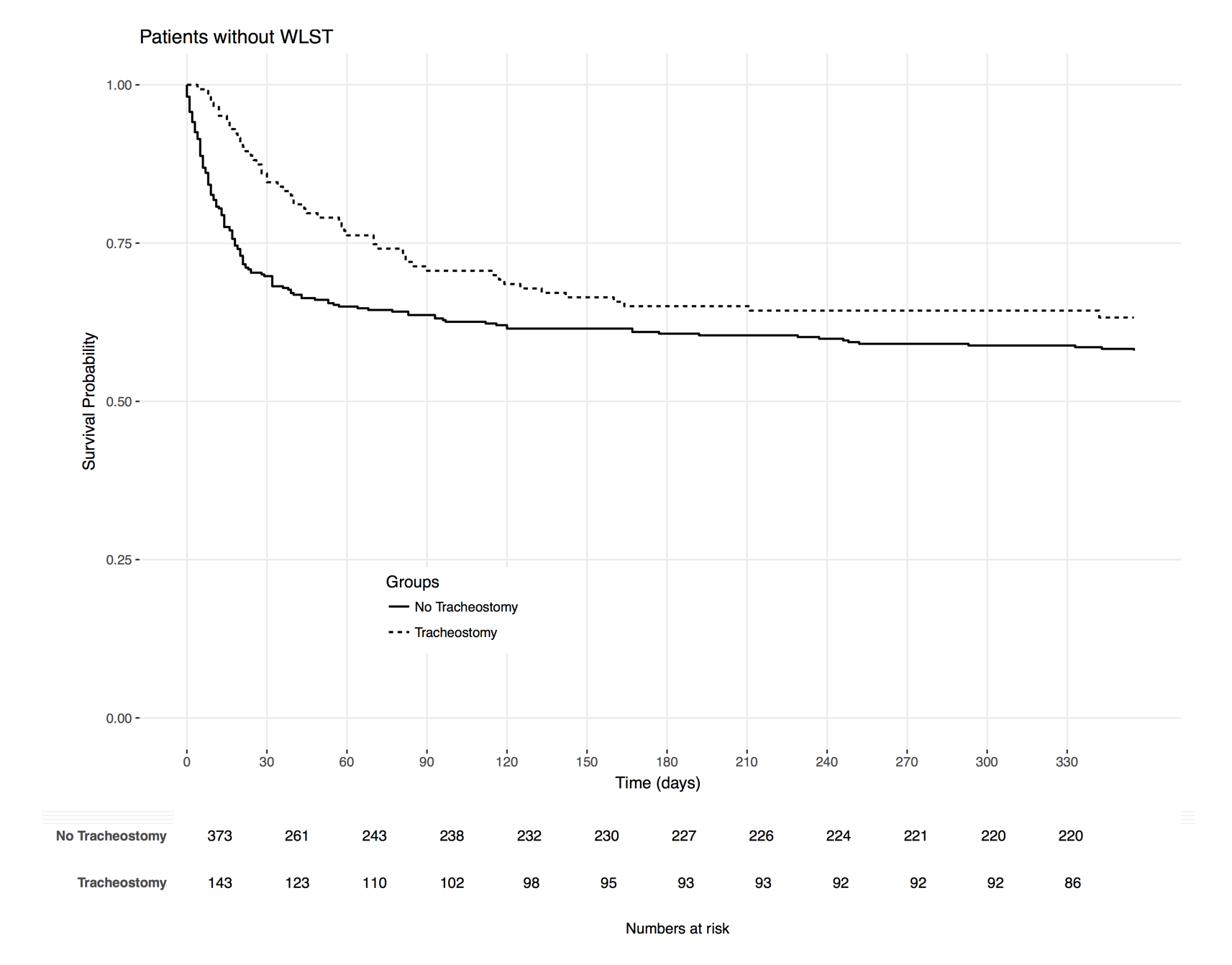

Supplement: S1 Fig — In the subgroup of patients without withdrawal of life sustaining therapies, there was no significant difference between patients with or without tracheostomy. Log-Rank test, p = 0.08. Cox regression model adjusted on age, SAPS II and diagnosis upon admission: HR CI95 0.74 [0.5–1.09], p = 0.1. (TIFF) [file pone.0220399.s001.tiff]
