# Supplementary material for: Tracheostomy and long-term mortality in ICU patients undergoing prolonged mechanical ventilation
Source: PLoS One. 2019 Oct 2;14(10):e0220399. doi: 10.1371/journal.pone.0220399 (PMC6774500; doi:10.1371/journal.pone.0220399)
Supplement: S1 Table — SAPS: Simplified Acute Physiological Score. RBC: red blood cell. (DOCX) [file pone.0220399.s002.docx]

**S1 Table.** Patients matched in the Tracheostomy group and in the prolonged MV group without tracheostomy

| Variables | Before Matching | |  | After Matching | |  |
| --- | --- | --- | --- | --- | --- | --- |
|  | No Tracheostomy  N=458 | Tracheostomy  N=157 | SD | No Tracheostomy  N=150 | Tracheostomy  N=150 | SD |
| Age | 65 [54-75] | 61 [50-69] | -26.1 | 62 [49-73] | 62 [52-70] | 3.4 |
| Gender | 297 (64.8) | 110 (70.1) | 11.1 | 101 (67.3) | 105 (70) | 5.7 |
| SAPS II | 51 [37-65] | 44 [31-59] | -26.7 | 45.5 [35-59.5] | 44.5 [32-60.5] | -2 |
| Charlson | 3 [1-5] | 3 [1-4] | -18.3 | 3 [1-4] | 3 [1-4] | 2.1 |
| Cause of admission |  |  |  |  |  |  |
| Neurologic | 62 (13.6) | 32 (20.4) | 18.2 | 28 (18.7) | 27 (18) | -1.7 |
| Acute Respiratory failure | 97 (21.2) | 21 (13.4) | -20.8 | 22 (14.7) | 21 (14) | -1.9 |
| In-ICU events |  |  |  |  |  |  |
| RBC Transfusion | 273 (59.6) | 106 (67.5) | 16.5 | 104 (69.3) | 101 (67.3) | -4.3 |
| Renal Replacement Therapy | 160 (34.9) | 62 (39.5) | 9.4 | 57 (38) | 61 (40.7) | 5.4 |
| Catecholamine | 381 (83.2) | 131 (83.4) | 0.7 | 125 (83.3) | 126 (84) | 1.8 |

Legend: SAPS : Simplified Acute Physiological Score. RBC: red blood cell
