# Supplementary material for: Tracheostomy and long-term mortality in ICU patients undergoing prolonged mechanical ventilation
Source: PLoS One. 2019 Oct 2;14(10):e0220399. doi: 10.1371/journal.pone.0220399 (PMC6774500; doi:10.1371/journal.pone.0220399)
Supplement: S2 Table — During the first year of follow-up, there was no statistically significant difference regarding the Mental and Physical components of the SF-36 between both groups (p = 0.9 and 0.9 respectively). There is no statistically significant difference in IES-R, Anxiety symptoms (HADS scale) or Depression symptoms (HADS scale) between the 2 groups (respectively p = 0.9, 0.2, 0.5), or evolution over time (respectively p = 0.1, p = 0.4, p = 0.3). Two-way ANOVA. (DOCX) [file pone.0220399.s003.docx]

**S2 Table.** Long-term quality of life in patients with or without tracheostomy

|  | Tracheostomy | | | No Tracheostomy | | |
| --- | --- | --- | --- | --- | --- | --- |
|  | 3 months  N=44 | 6 months  N=44 | One year  N=38 | 3 months  N=53 | 6 months  N=53 | One year  N=48 |
| SF-36 |  |  |  |  |  |  |
| Physical Component Score | 38 (±22) | 45 (±26) | 49 (±22) | 41 (±21) | 46 (±24) | 51 (±26) |
| Mental Component Score | 45 (±23) | 51 (±26) | 59 (±26) | 46 (±22) | 50 (±24) | 53 (±23) |
| HADS |  |  |  |  |  |  |
| Anxiety | 8 (±5) | 7 (±4) | 6 (±4) | 7 (±5) | 8 (±5) | 7 (±4) |
| Depression | 7 (±4) | 7 (±5) | 6 (±4) | 7 (±4) | 7 (±5) | 7 (±4) |
| IES-R | 21 (±18) | 21 (±18) | 18 (±15) | 25 (±19) | 19 (±18) | 15 (±15) |

Legend: During the first year of follow-up, there was no statistically significant difference regarding the Mental and Physical components of the SF-36 between both groups (p=0.9 and 0.9 respectively). There is no statistically significant difference in IES-R, Anxiety symptoms (HADS scale) or Depression symptoms (HADS scale) between the 2 groups (respectively p=0.9, 0.2, 0.5), or evolution over time (respectively p=0.1, p=0.4, p=0.3). Two-way ANOVA.
